# Kinetic Estimation of GFR Improves Prediction of Dialysis and Recovery after Kidney Transplantation

**DOI:** 10.1371/journal.pone.0125669

**Published:** 2015-05-04

**Authors:** Timothy J. Pianta, Zoltan H. Endre, John W. Pickering, Nicholas A. Buckley, Philip W. Peake

**Affiliations:** 1 Prince of Wales Clinical School, University of New South Wales, Sydney, Australia; 2 Melbourne Medical School, University of Melbourne, Melbourne, Australia; 3 Department of Medicine, University of Otago, Christchurch, New Zealand; 4 Clinical Pharmacology, University of Sydney, Sydney, Australia; Mario Negri Institute for Pharmacological Research and Azienda Ospedaliera Ospedali Riuniti di Bergamo, ITALY

## Abstract

**Background:**

The early prediction of delayed graft function (DGF) would facilitate patient management after kidney transplantation.

**Methods:**

In a single-centre retrospective analysis, we investigated kinetic estimated GFR under non-steady-state conditions, KeGFR, in prediction of DGF. KeGFR_sCr_ was calculated at 4h, 8h and 12h in 56 recipients of deceased donor kidneys from initial serum creatinine (sCr) concentrations, estimated creatinine production rate, volume of distribution, and the difference between consecutive sCr values. The utility of KeGFR_sCr_ for DGF prediction was compared with, sCr, plasma cystatin C (pCysC), and KeGFR_pCysC_ similarly derived from pCysC concentrations.

**Results:**

At 4h, the KeGFR_sCr_ area under the receiver operator characteristic curve (AUC) for DGF prediction was 0.69 (95% CI: 0.56–0.83), while sCr was not useful (AUC 0.56, (CI: 0.41–0.72). Integrated discrimination improvement analysis showed that the KeGFR_sCr_ improved a validated clinical prediction model at 4h, 8h, and 12h, increasing the AUC from 0.68 (0.52–0.83) to 0.88 (0.78–0.99) at 12h (p = 0.01). KeGFR_pCysC_ also improved DGF prediction. In contrast, sCr provided no improvement at any time point.

**Conclusions:**

Calculation of KeGFR from sCr facilitates early prediction of DGF within 4 hours of renal transplantation.

## Introduction

Calculation of a non-steady state (kinetic) glomerular filtration rate, the KeGFR, has recently been advocated in assessment of acute kidney injury (AKI) and renal recovery [1]. The formula is derived from the change in consecutive values of serum creatinine (sCr), and the estimated creatinine production rate and volume of distribution (V_d_) to estimate GFR. This approach to characterising clearance is adaptable to alternative circulating filtration biomarkers including cystatin C (CysC) [1].

Twenty to thirty percent of deceased donor kidneys and approximately half of kidneys donated after cardiac death develop delayed graft function (DGF), defined as requirement for dialysis within the first week after transplantation [2]. DGF is associated with increased rates of rejection and graft loss, inferior graft function and increased length of hospital stay [3]. Early identification of patients with DGF is already important in modifying standard immunosuppressive and antimicrobial (cytomegalovirus and *Pneumocystis jirovecii*) prophylaxis after transplantation. Thus avoidance or reduction in the dose of standard but potentially nephrotoxic immunosuppressive therapy with calcineurin-inhibitors, tacrolimus or cyclosporine, and a reduction in doses of valgan- or vala-ciclovir and of co-trimoxazoles is essential in DGF. In addition, while there have been no clinical studies demonstrating successful intervention to prevent acute tubular injury or accelerate recovery so far, rapid identification of DGF may facilitate intervention when appropriate agents eventually become available [4,5]. However, current methods for prediction of DGF after kidney transplantation are unreliable.

While the presence of anuria is a specific sign of DGF, it is not sensitive [6], and change in sCr is an unreliable marker of DGF for the first 24 hours after transplantation [5,7]. Immediately after transplantation, sCr is influenced by many factors including residual renal function, pre-transplant dialysis and peri-operative fluid administration [4] and is known to be a poor predictor of DGF [7]. A clinical prediction model has been developed with an online calculator to predict DGF from donor-, recipient-, and transplant-related variables [8]. While this model has been validated [9], its diagnostic utility for predicting DGF in two cohorts has been only “fair” [10] with areas under the receiver operator characteristic curve (AUCs) of 0.70 [8] and 0.71 [9]. This risk assessment is fixed once transplantation has occurred, so injury signals after transplantation are not incorporated and cannot enhance prediction of DGF.

An alternative circulating filtration marker is plasma cystatin C (pCysC). pCysC is produced by all nucleated cells at a near constant rate, does not re-circulate after glomerular filtration nor undergo tubular secretion [11]. While pCysC is subject to similar perioperative influences as sCr, CysC is only distributed in extracellular fluid (ECF) [4,11,12], rather than total body water (TBW). With one-third the V_d_ of creatinine, pCysC reaches a new steady state three times more rapidly than sCr after GFR is perturbated [4]. Several studies have recognized that pCysC outperforms sCr for DGF prediction and advocated for pCysC to replace sCr [7,13].

Using sCr [14] or pCysC [15] under steady-state conditions GFR can be estimated using formulae that adjust for age, race and sex. The Cockroft-Gault formula [16] adjusts for age, sex and weight to estimate creatinine clearance (CrCl). However, these formulae and the classic equation for CrCl are invalid under non-steady state conditions such as occur after transplantation [4]. CysC clearance cannot be measured in healthy kidneys directly because of tubular uptake and lysosomal metabolism of filtered CysC [4,11,17].

We hypothesised that calculation of KeGFR would improve the prediction of DGF independently of a validated clinical risk prediction model [8]. We also hypothesised that pCysC and KeGFR derived from pCysC would predict DGF better than sCr-based estimates. These hypotheses were evaluated in a retrospective analysis of prospectively studied renal transplant recipients in which calculation of KeGFR from sCr facilitated early prediction of DGF within 4 hours of renal transplantation.

### Subjects and Methods

The study was conducted under institutional Human Ethics Committee approval (EC00134:10/113), in adherence to the Declaration of Helsinki and the principles outlined in the ‘Declaration of Istanbul on Organ Trafficking and Transplant Tourism’. Specifically, none of the transplant donors were from a vulnerable population and all donors or next of kin provided written informed consent that was freely given.

We performed a retrospective analysis of previously published prospectively acquired sCr and pCysC data [6]. Consecutive eligible patients aged 18 years or older undergoing deceased donor kidney transplantation between 2011 and 2013 at Prince of Wales Hospital, Sydney were approached for inclusion. Clinical decisions regarding patient treatment, including dialysis were made independently by treating physicians. Patients received a uniform protocol of corticosteroids, basiliximab, and mycophenolate sodium, and tacrolimus or cyclosporine at the treating physician’s discretion.

### Samples

sCr was measured immediately after organ reperfusion as part of routine care. Additional blood samples were collected at 4h, 8h and 12h after organ reperfusion for the study. Blood was immediately centrifuged and serum and plasma was aliquoted and stored at -80°C prior to batched assay.

### Assays

sCr was measured using enzymatic methods on an automated chemical analyser (Konelab 20XT, Thermo Fisher Scientific, Waltham, MA, USA) according to manufacturer’s recommendations. CysC was measured using microparticle-enhanced immunoturbidometric methods using the same analyser and a calibrator (X791201, Dako, Glostrup, Denmark) traceable to the International Federation of Clinical Chemistry Working Group certified reference material (ERM-DA471/IFCC) [18].

### Variables examined

The primary outcome was DGF, defined as the need for dialysis within 1 week of transplantation [3]. The predictive utility of sCr, pCysC, unadjusted (steady-state) eGFR derived from sCr and the Chronic Kidney Disease Epidemiology Collaboration (CKD-EPI) formula [14] (eGFR_sCr_), unadjusted eGFR derived from pCysC and the CKD-EPI formula [15] (eGFR_pCysC_), and KeGFR using sCr (KeGFR_sCr_) for DGF was assessed at 4h, 8h and 12h, and KeGFR using pCysC (KeGFR_pCysC_) assessed at 8 and 12h.

KeGFR was calculated using the formula [1]:

$$KeGFR\;=\;\frac{B_{C}\;\times \;eGFR}{MeanB_{c}}\;\times \;\left(1-\frac{24\;\times \;\Delta B_{C}}{\Delta t\;\times \;Max\Delta B_{C}/d}\right)$$

B_c_ × eGFR is the circulating biomarker concentration (B_c_, sCr or pCysC) multiplied by the corresponding unadjusted eGFR [14,15]; MeanB_c_ is the mean of two consecutive values of B_c_ (e.g. at 4h and 8h); ΔB_c_ is the difference between each two values; Δt is the interval between samples (hours), and MaxΔB_c_/d is the maximal theoretical increase in sCr or pCysC in 1 day when GFR is zero.

Two values must be determined: i) B_c_×eGFR and ii) MaxΔB_c_/d. Assuming mass balance principles [1,19] these 2 terms are closely related [1]:

- B_c_×eGFR is proportional to daily circulating biomarker production, and
- MaxΔB_c_/d equals daily circulating biomarker production divided by V_d_,

To convert B_c_×eGFR [e.g. sCr (μmol/L) × eGFR (mL/min/1.73m^2^)] to daily circulating biomarker production (μmol/d/1.73m^2^) a conversion factor of 1.44 accounted for the 1440 minutes per day and 1000 millilitres per litre. B_c_×eGFR is expressed in μmol/d/1.73m^2^ or mg/d/1.73m^2^ and KeGFR is expressed in mL/min/1.73m^2^, however to avoid unwieldy terms, we have omitted further referring to body surface area in subsequent discussion.

The CKD-EPI formula was used for eGFR estimation using both creatinine [14] and CysC [15], i.e., in both the “B_c_×eGFR” and “MaxΔB_c_/d” terms. As suggested [1] B_c_×eGFR was calculated once for the entire acute renal episode and, for consistency, the 4h value of sCr or pCysC was used. Thus,

$$Daily\;circulating\;biomarker\;production\;=\;B_{c}\left(4h\right)\;\times \;eGFR\;\times \;1.44$$

and

$$Max\Delta Bc/d\;=\;B_{c}\left(4h\right)\;\times \;eGFR\;\times \;1.44/V_{d}$$

For sCr, estimated V_d_ = 0.6 × body weight (kg) [1,20]. For pCysC, estimated V_d_ = 0.2 × body weight (kg) [12].

Because eGFR cannot be less than 0, a value of 0 was assigned to any negative calculated value of KeGFR.

### Statistical analysis

Analyses were conducted with Prism v6.0 (GraphPad, La Jolla, CA) and MATLAB 2012b (Mathworks, Natick, MA). Because dialysis modifies sCr and pCysC, after initiation of dialysis, patients were excluded from analysis at subsequent timepoints.

Performance in prediction of DGF was assessed from the AUC calculated at each timepoint and comparisons made using the DeLong method [21]. Optimal cut-offs were values with the maximal Youden index [22]. KeGFR represents the arithmetic mean of estimated GFR between two timepoints. For example, the KeGFR_sCr_ calculated at 4h most closely estimated eGFR at 2h (i.e. the midpoint between 0h and 4h). Since the second value of sCr required for this calculation was unavailable until 4h, its performance was evaluated against the 4h sCr. Agreement between different eGFR and KeGFR estimates was assessed using Bland-Altman analysis.

The baseline model used the published [8] and validated [9] risk prediction model for DGF. Individual DGF risk was calculated online (http://www.transplantcalculator.com) after entering 9 recipient-, 8 donor-, and 3 transplantation-related factors as described [8] (Table 1). Integrated discrimination improvement (IDI) analysis was used to determine the enhancement added by each variable to this model. Multivariable logistic regression with forward entry was used to construct models by alternately adding variables to the reference score and calculating the probability of DGF for each patient. IDIs were calculated for each variable to determine the mean increase in calculated risk for those who developed DGF (IDI-DGF), and reduction for those who did not (IDI-non-DGF) after the addition of the variable to the baseline score [23].

**Table 1:** Baseline characteristics of transplant recipients and donors.

Total, n = 56	Non-DGF, n = 34	DGF, n = 22	p value
**Recipient Characteristics**			
**Male** ^a^ **, *n (%)***	21 (62)	15	0.78
**Age** ^a^ **, *years***	56 (49–62)	50 (47–62)	0.76
**Age < 16 years** ^b^	0	0	
**Ethnicity, *n (%)***			
**Black African** ^a^	0	0	
**Caucasian**	22 (65)	17 (77)	0.38
**Non-Caucasian**	12 (35)	5 (23)	
**Asian**	10 (29)	2 (9)	
**Pacific Islander**	2 (6)	1 (4)	
**Other**	0	2 (9)	
**Body mass index (kg/m2)** ^a^ **, *median (IQR)***	25 (23–28)	30 (25–33)	0.05
**% Peak PRA** ^a^ **, *median (IQR)***	3 (1–11)	10 (3–39)	0.12
**Duration of dialysis** ^a^ **(months), *median (IQR)***	64 (31–84)	75 (35–91)	0.46
**Previous transplant** ^a^ **(yes), *n (%)***	3 (8)	5 (23)	0.24
**Pretransplant transfusion** ^a^ **(yes), *n (%)***	7 (21)	6 (27)	0.74
**Diabetes mellitus** ^a^ **, *n (%)***	5 (14)	6 (27)	0.31
**Extrarenal transplant** ^b^ **, *n***	0	0	
**Hemodialysis** ^c^	28 (82)	20 (91)	0.46
**Induction regimen**			
**Calcineurin Inhibitor**			
**Tacrolimus**	26 (74)	17 (81)	1.00
**Cyclosporine A**	8 (24)	5 (23)	
**Deceased Donor Characteristics**	**n = 34** ^d^	**n = 22** ^d^	
**Male, n (%)**	18 (53)	11 (50)	1.00
**Age** ^a^ **, years (IQR)**	54 (41–65)	56 (43–61)	0.78
**Cardiac Death** ^a^ **, *n (%)***	2 (6)	9 (41)	0.002
**ECD, *n (%)***	8 (23)	3 (14)	0.50
**Terminal sCr** ^a^ **, *μmol/L (IQR)***	81 (60–95)	61 (56–82)	0.10
**Cause of death, *n (%)***			
**Stroke** ^a^	14 (41)	10 (45)	0.79
**Anoxia** ^a^	3 (9)	2 (9)	1.00
**Transplant Characteristics**			
**HLA mismatches** ^a^ **, median (IQR)**	4 (2–5)	4 (2–6)	0.54
**Ischaemia time (min), *median (IQR)***			
**Cold** ^a^	531 (408–787)	708 (514–997)	0.04
**Warm** ^a^	43 (34–52)	47 (32–67)	0.68
**Machine Perfusion** ^b^	0	0	

Key:

^a^: all others peritoneal dialysis

^b^: all others rabbit anti-thymocyte globulin

^c^: No donor contributed a kidney to more than one recipient within the cohort

DGF: Delayed Graft Function, Non-DGF: Non-Delayed Graft Function,

ESKD: End Stage Kidney Disease; ECD: Expanded Criteria Donor; sCr: serum creatinine.

Sensitivity analyses evaluated alternative assumptions of creatinine and CysC production. (S2 Table). MaxΔsCr/d was alternatively estimated using the Cockroft-Gault formula [16], and a fixed value (235 mmol/L/d, i.e. 3mg/dL/d a moderately high estimate of MaxΔsCr/d in anuric AKI) [1,24] for all patients, while MaxΔpCysC/d was alternatively estimated using the Sjostrom formula [12], and a fixed value (3 mg/L/d) [1] (S2 Table). Expression of KeGFR in mL/min (i.e. KeGFR × BSA / 1.73) was also evaluated (S3 Table). The inclusion of recipients of live-donor kidneys concurrently recruited was assessed in a further sensitivity analyses (S4 Table).

## Results

56 recipients of deceased-donor kidney transplants were consented for inclusion. Patient characteristics are shown in Table 1. After transplantation, four patients commenced dialysis between 4h and 8h, leaving 52 patients for analysis at 8h and 12h (Table 2). No patient was dialysed for isolated hyperkalaemia and there were no cases of primary non-function.

**Table 2:** Prediction of DGF using KeGFR versus the clinical model.

	Variables	AUC (95% CI)	p	IDI-DGF (95% CI)	IDI-Non-DGF (95% CI)
***Base Model 4h (n = 56)***		0.72 (0.58 to 0.86)		**a**	**a**
***Base Model +***	**sCr**	0.73 (0.59 to 0.87)	0.79	0.01 (-7 x 10^–4^ to 0.07)	0.01 (-4 x 10^–4^ to 0.05)
***Base Model +***	**KeGFR**_**sCr**_	0.77 (0.63 to 0.89)	0.24	0.03 (1 x 10^–6^ to 0.11)^b^	0.02 (3 x 10^–5^ to 0.08)^b^
***Base Model +***	**pCysC**	0.77 (0.64 to 0.90)	0.13	0.02 (-5 x 10^–5^ to 0.11)	0.02 (-1 x 10^–5^ to 0.08)
***Base Model 8h & 12h (n = 52)***		0.68 (0.52 to 0.83)			
***8h (n = 52)***					
***Base Model +***	**sCr**	0.67 (0.52 to 0.83)	0.39	0.00 (-0.004 to 0.004)	0.00 (-0.001 to 0.002)
***Base Model +***	**KeGFR**_**sCr**_	0.81 (0.67 to 0.94)	0.16	0.07 (9 x 10^–4^ to 0.21)^b^	0.04 (6 x 10^–4^ to 0.12)^b^
***Base Model +***	**pCysC**	0.78 (0.65 to 0.92)	0.10	0.03 (-5 x 10^–4^ to 0.14)	0.02 (-2 x 10^–4^ to 0.09)
***Base Model +***	**KeGFR**_**pCysC**_	0.78 (0.64 to 0.92)	0.19	0.06 (2 x 10^–4^ to 0.20)^b^	0.04 (0.15 to 0.12)^b^
***12h (n = 52)***					
***Base Model +***	**sCr**	0.71 (0.56 to 0.86)	0.32	0.01 (-0.004 to 0.06)	0.00 (-0.001 to 0.03)
***Base Model +***	**KeGFR**_**sCr**_	0.88 (0.78 to 0.99)	0.01^b^	0.18 (0.04 to 0.35)^b^	0.10 (0.03 to 0.21)^b^
***Base Model +***	**pCysC**	0.80 (0.67 to 0.93)	0.11	0.06 (-8 x 10^–4^ to 0.19)	0.03 (-2 x 10^–4^ to 0.11)
***Base Model +***	**KeGFR**_**pCysC**_	0.82 (0.69 to 0.95)	0.09	0.11 (0.02 to 0.25)^b^	0.06 (0.01 to 0.15)^b^

Model enhancement was analysed by calculation of the IDI. The clinical base model was derived from recipient-, donor- and transplant related factors (reference [8]). There is no KeGFRpCysC at 0–4h since no 0h pCysC data were available.

Key:

^a^: metrics are not calculable for baseline model alone

^b^: p < 0.05 vs. base model; IDI: integrated discrimination improvement.

### Circulating biomarkers, eGFR and KeGFR: univariable analysis

**Fig 1:**
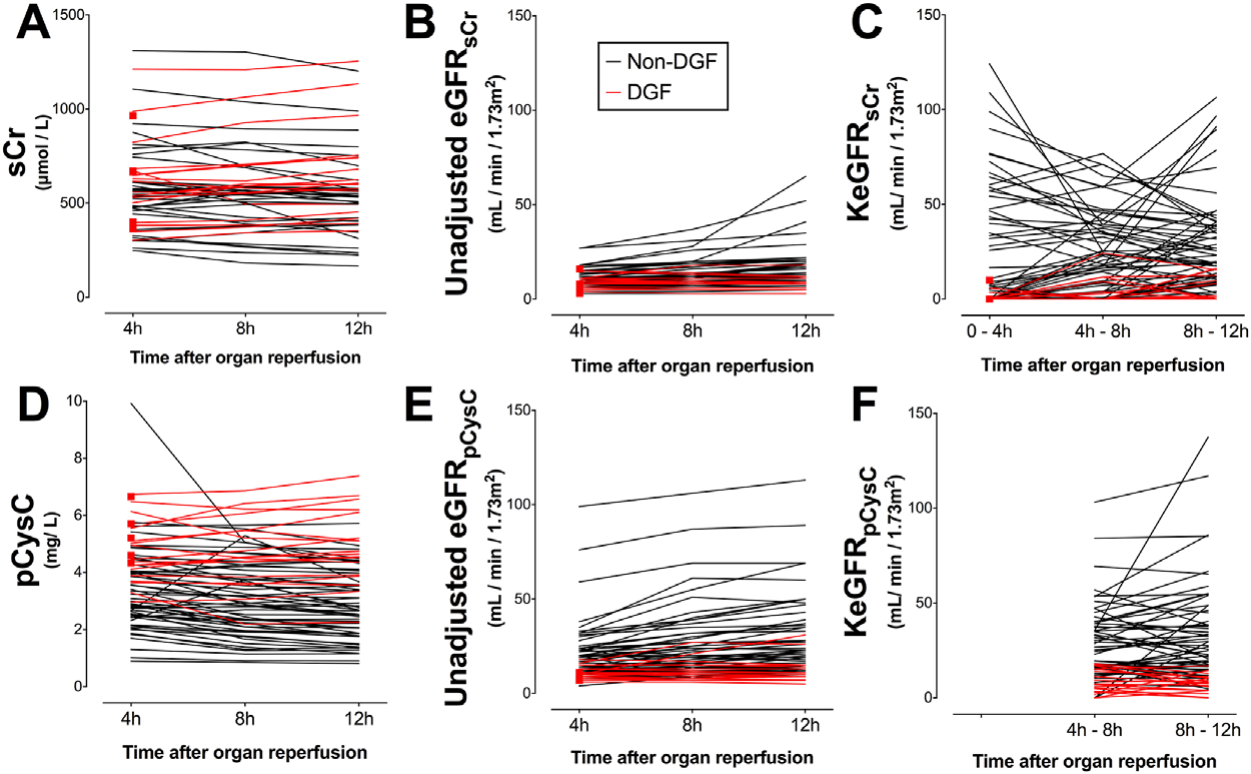
Serum creatinine, plasma cystatin C, unadjusted eGFR and kinetic estimated GFR for each patient. Values for each patient of (A) serum creatinine (sCr), (B) unadjusted estimate of GFR using sCr (eGFR_sCr_), (C) kinetic estimate of GFR using sCr (KeGFR_sCr_), (D) plasma cystatin C (pCysC), (E) unadjusted estimate of GFR using pCysC (eGFR_pCysC_), and (F) kinetic estimate of GFR using pCysC (KeGFR_pCysC_). Results are stratified for patients developed delayed graft function, i.e. dialysis requirement within 7 days (red) and those who did not (black). Red squares represent patients who were dialysed before the next timepoint. There is no KeGFR_pCysC_ at 0–4h since no 0h pCysC data were available.

**Fig 2:**
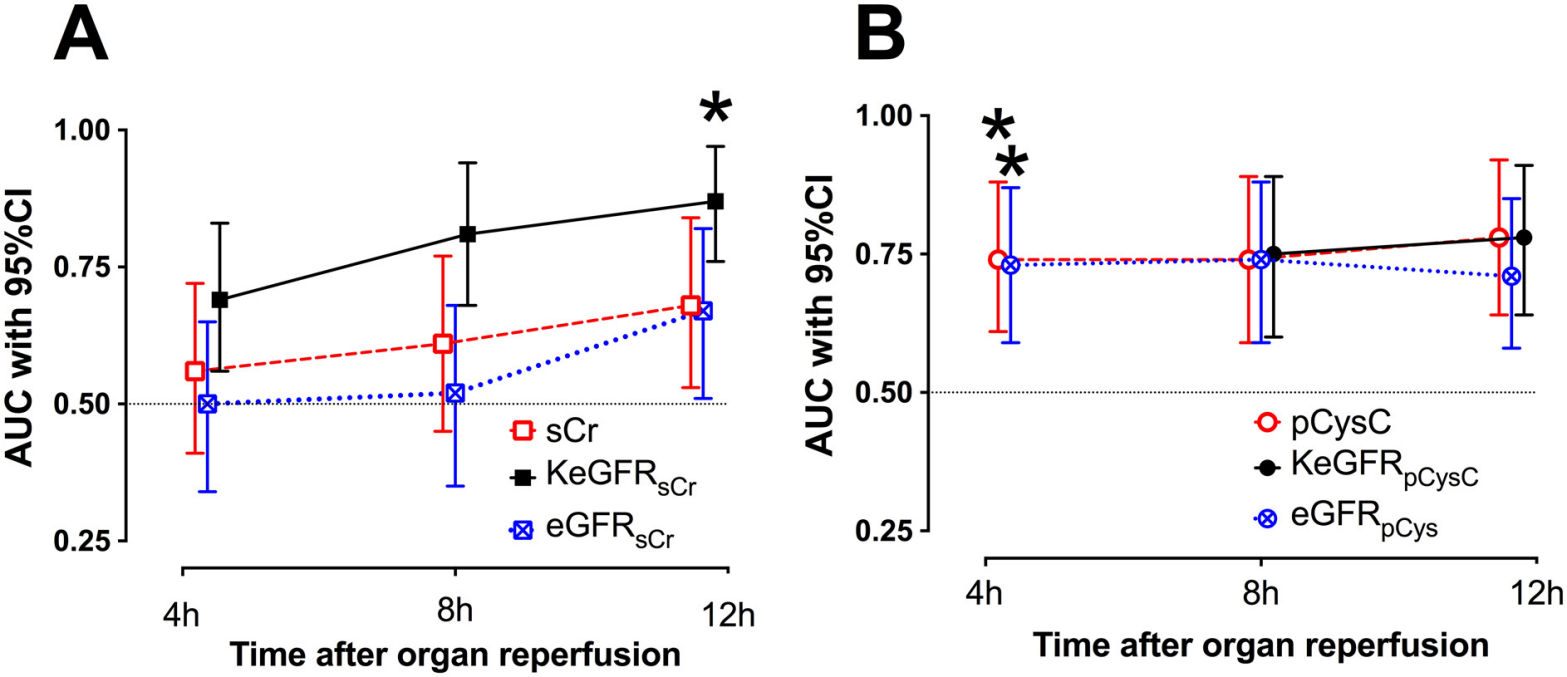
KeGFR versus eGFR, and KeGFR_sCr_ vs. KeGFR_pCysC_. A—C: Unadjusted eGFR_sCr_ and KeGFR_sCr_ values stratified for DGF (red) or Non-DGF (black) at 4h (A), 8h (B), and 12h (C). D: Bland-Altman Plot comparing eGFR and KeGFR at 4h (blue triangles), 8h (open squares), and 12h (red circles) after kidney transplantation. Dotted line represents bias (mean difference between parameters); dashed lines represent 95% limits of agreement. E and F: Values for each patient of eGFR_pCysC_ and KeGFR_pCysC_ stratified for DGF or Non-DGF at 8h (E) and 12h (F) G: Bland-Altman Plot comparing eGFR and KeGFR at 8h and 12h after kidney transplantation. H: Bland-Altman Plot comparing KeGFR_sCr_ and KeGFR_pCysC_ at 8h and 12h after kidney transplantation. There was no KeGFR_pCysC_ determined at 4h since no 0h pCysC data were available. #: p ≥ 0.05, * p < 0.05.

**Fig 3:**
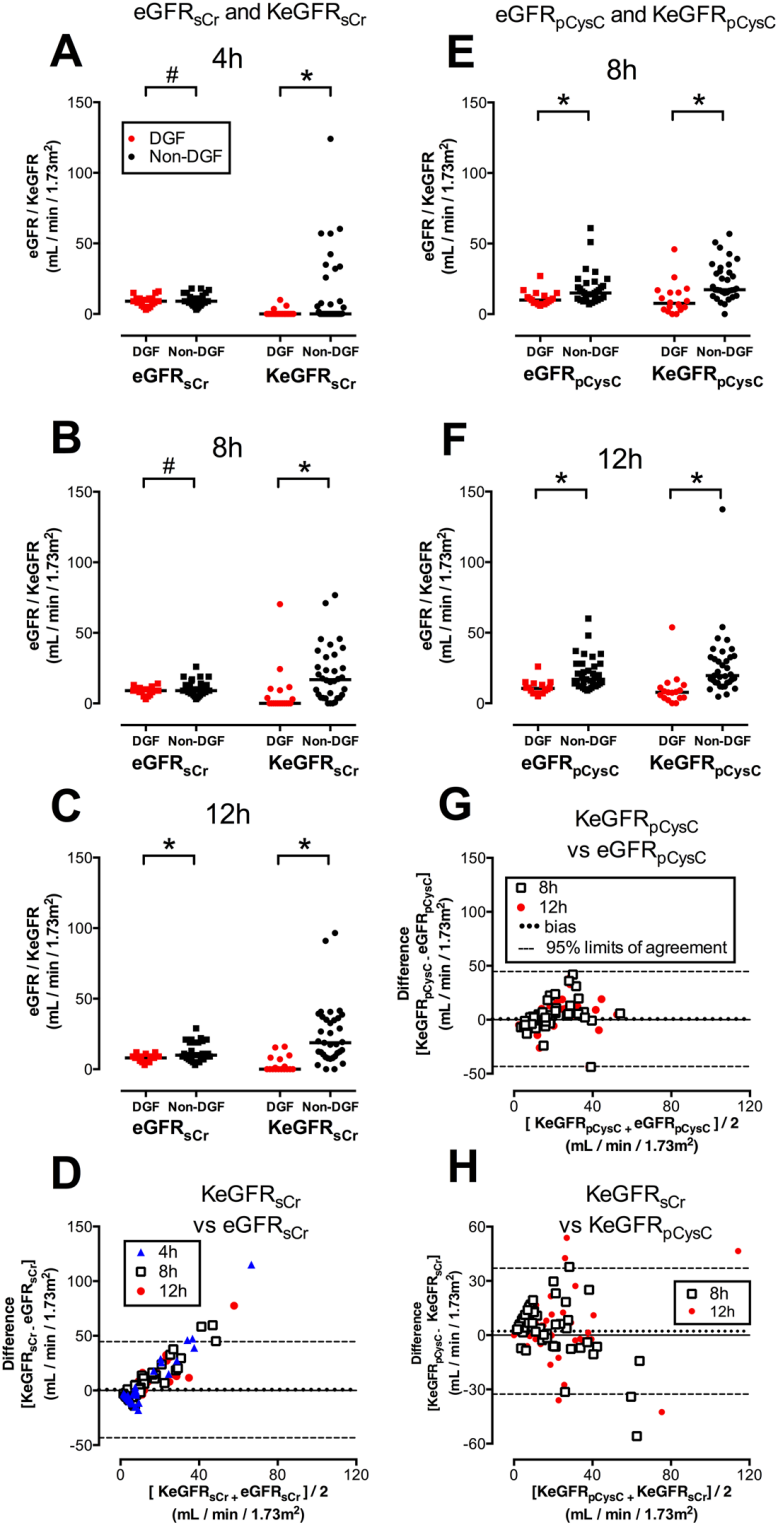
KeGFR prediction of DGF compared with unadjusted eGFR and sCr. A: estimated kinetic GFR using serum creatinine (KeGFR_sCr_) compared with serum creatinine (sCr) and unadjusted estimate of GFR using sCr (eGFR_sCr_). Figures show area under receiver operator characteristic curve (AUC) and 95% confidence intervals for prediction of DGF at 4h (n = 56), 8h (n = 52) and 12h (n = 52). Asterisk (*) denotes p < 0.05 for the comparison with the AUC for sCr at the same time.

sCr, pCysC, eGFR_sCr_, eGFR_pCysC_, KeGFR_sCr_, and KeGFR_pCysC_ for each patient are presented in Fig 1; the latter four are compared in Fig 2. AUCs for prediction of DGF are presented in Fig 3.

KeGFR_sCr_ predicted DGF at all time points with higher AUC values than sCr, and these differences were statistically significant at 12h (Fig 3A).

pCysC also predicted DGF at all time points with higher AUC values than sCr; the difference significant only at 4h (Fig 3B).

KeGFR_pCysC_ predicted DGF with higher AUC values than sCr at both 8h and 12h but the improvement was not significant at either time. There was no significant difference between KeGFR_pCysC_ and pCysC at either 8h or 12h (Fig 3B).

eGFR_sCr_ performed no better for prediction of DGF than sCr alone (Fig 3A), and eGFR_pCysC_ performed no better for prediction of DGF than pCysC alone at any time (Fig 3B).

### Integrated discrimination improvement (IDI) analysis

**Fig 4:**
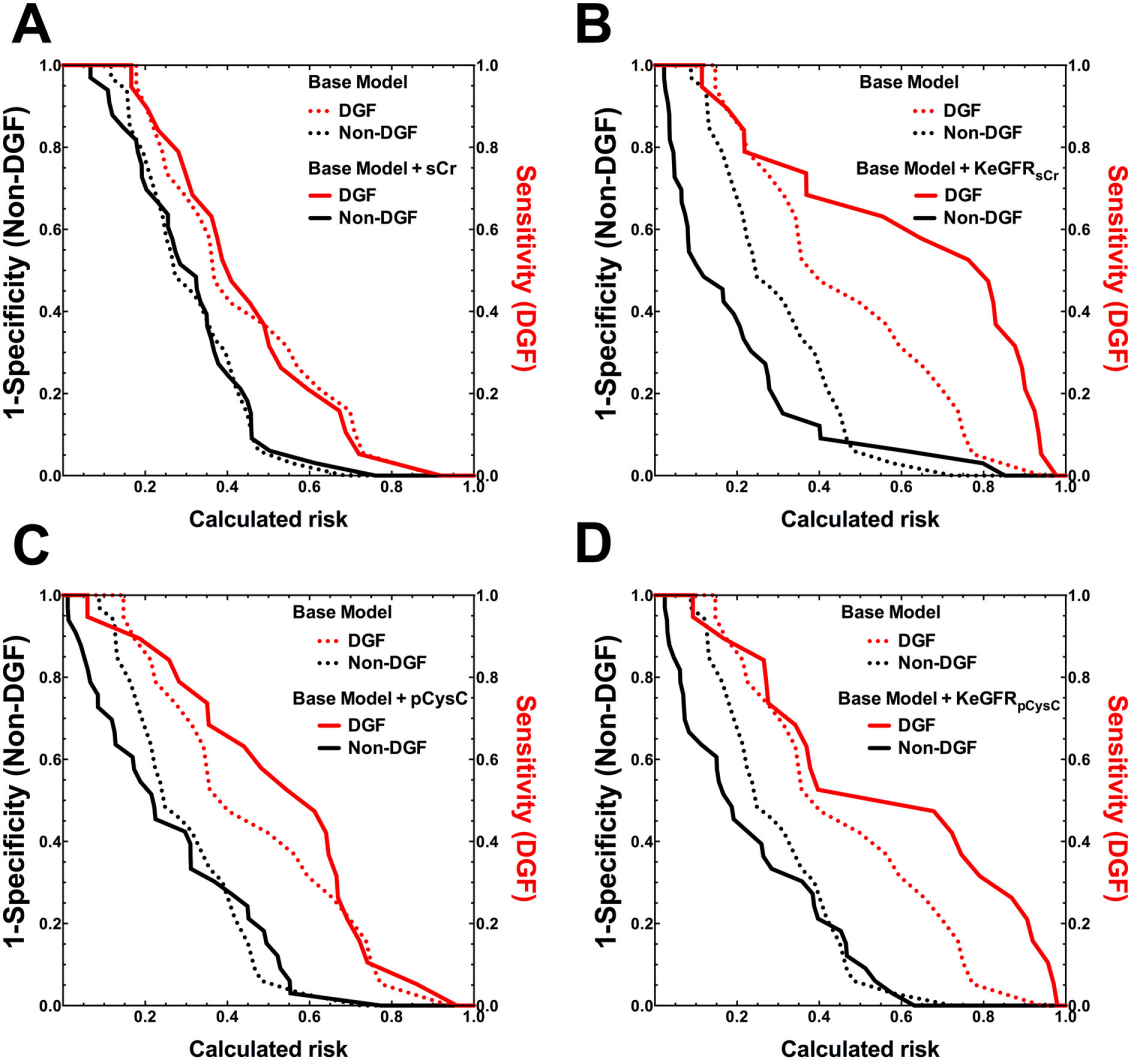
Clinical model enhancement by measured and calculated variables at 12h. A: serum creatinine (sCr), B: estimated GFR using sCr (KeGFR_sCr_) C: plasma Cystatin C (pCysC), D: estimated GFR using pCysC (KeGFR_pCysC_).

Incorporation of KeGFR_sCr_ independently improved prediction of DGF at 4h [average increase in calculated risk of DGF (IDI-DGF): 0.03 (95% CI: 1 x 10^–6^ to 0.11)] and non-DGF [IDI-non-DGF: 0.02 (95% CI: 3 x 10^–5^ to 0.08)] (Table 2) with the AUC for the new model increasing from 0.72 to 0.77 (95% CI: 0.63–0.89). Enhancement of the clinical model with KeGFR_sCr_ was also seen at 8h and 12h (Table 2, Fig 4).

Risk assessment plots (see reference [22]) for the base model, (dotted lines) and models after addition of variables (solid lines). Red lines are sensitivity versus the calculated risk for patients who developed delayed graft function. Black lines represent 1-specificity versus the calculated risk for those who did not have delayed graft function. Improved risk assessment is demonstrated by movement of the red curve to the top-right corner and black curve to the bottom left corner after addition of a biomarker.

KeGFR_pCysC_ performed similarly to KeGFR_sCr_ improving the prediction of DGF at both 8h and 12h (Table 2, Fig 4) although the quantum of improvement at each time was less than seen for KeGFR_sCr_.

After adjusting for the baseline score, IDI analysis showed that sCr did not improve the prediction of DGF at any time (Table 2, Fig 4).

Although univariable analysis showed better performance by pCysC than sCr, pCysC did not independently improve DGF prediction at any time (Table 2, Fig 4).

### Comparison of KeGFR_sCr_ and KeGFR_pCysC_

KeGFR_pCysC_ was generally higher than KeGFR_sCr_ at both 8h and 12h (Fig 2H). Optimal cut-off points for the prediction of DGF for KeGFRsCr at 4h, 8h and 12h were, respectively 0, 4, and 5 mL/min/ 1.73 m^2^ (S1 Table). The optimal cut-off points were higher for KeGFR_pCysC_ than KeGFR_sCr_ (S1 Table).

### Sensitivity analyses

The predictive performance of KeGFR_sCr_, and of KeGFR_pCysC_, were robust to changes in the methods for calculating MaxΔB_c_/d, with no significant differences in AUCs (S2 Table). The main impact was to change modestly the optimal cut-offs of KeGFR for predicting DGF. Despite a difference in BSA between DGF and non-DGF patients (Table 1), expression of KeGFR in mL/min similarly produced no significant difference in AUCs (S2 Table). However, the apparent performance of all biomarkers including KeGFR_sCr_ was improved by inclusion of 25 recipients of live-donor kidneys (S4 Table).

### Long-term correlation

KeGFR_sCr_ at 12h was correlated to eGFR at 12 months (Spearman r = 0.30, p = 0.04). sCr, pCysC, eGFR_sCr_, eGFR_pCysC_, and KeGFR_pCysC_ were not significantly correlated to 12-month eGFR.

## Discussion

This study assesses the use of this novel method of estimating GFR under non-steady state conditions [1] and demonstrates that the KeGFR formula predicts DGF within 4 hours of renal transplantation. While the derivation of the formula has been explained elsewhere,[1], it has not previously been evaluated in any patient cohort. The unadjusted eGFR did not improve prediction of DGF, while AUCs for KeGFR_sCr_ were greater than for sCr. Since comparing AUCs has limited power to assess relative clinical utility [23,25], IDI analysis was used to evaluate the added value KeGFR_sCr_ provided over the baseline model [23]. In contrast to sCr alone [23], KeGFR_sCr_ independently enhanced the clinical model for DGF prediction at 4h, 8h, and 12h after transplantation and appears useful for early assessment of graft function.

The utility of pCysC and KeGFR_pCysC_ was similarly evaluated. Consistent with previous reports [7,13], AUCs for pCysC were fair [10] in transplant recipients and generally greater than sCr. After accounting for the baseline model, pCysC did not significantly improve DGF prediction. Although KeGFR_pCysC_ was only evaluated at 8h and 12h, performance was similar to KeGFR_sCr_.

While, for example, the cut-off of KeGFR_sCr_ that predicted DGF at 4h, 8h or 12h with 90% sensitivity lay between 0 and 7 mL/min/1.73m^2^ (S1 Table) the ideal diagnostic cut-off for any test requires prospective evaluation, and depends on the relative importance of identifying true positives (sensitivity) or true negatives (specificity), balancing the potential harms and benefits of any consequent intervention.

Applying the KeGFR formula did not further improve DGF prediction for pCysC as much as it did for sCr. At least three explanations are possible. Firstly, pCysC is less dependent on sex, race and body mass than sCr [26], thus unadjusted values require less “improvement” for clinical utility. Secondly, pCysC values approach steady-state more rapidly than sCr, reflected in the better agreement between KeGFR and eGFR for CysC than sCr at all timepoints. Thirdly, extra-renal clearance of biomarkers affects estimated GFR most at low GFRs, such as seen here. Extra-renal clearance has been estimated at 22mL/min/1.73m^2^ for CysC [12]_,_ versus <10mL/min/1.73m^2^ for creatinine [27].

KeGFR_sCr_ has several advantages over alternative approaches for identifying DGF. Firstly, sCr is inexpensive, routinely measured post-transplant, and familiar to all. The formula is relatively simple, calculation of KeGFR at any time requires only 2 values of sCr or pCysC. Indeed, the entire analysis presented here rests upon 4 values of sCr and 3 values of pCysC for each patient. However, while most physicians evaluate post-operative changes in sCr, the approach to changing sCr values is *ad hoc*. Chen proposed that use of the KeGFR formula in AKI could standardise the approach to changing sCr values adding “a quantitative and visual dimension to assessment of kidney function” [1]. Secondly, while sCr alone, and relative and absolute change in sCr are inadequate for early DGF prediction [5,6], the KeGFR_sCr_ formula reconciles the absolute value of sCr with rate of change to estimate kidney function. Thus, within the first half of the kinetic formula, “$\frac{B_{C}\;\times \;eGFR}{MeanB_{c}}$”, higher mean circulating biomarker concentrations produce lower KeGFR values. Within the second half, “$\left(1-\;\frac{24\;\times \;\Delta B_{C}}{\Delta t\;\times \;Max\Delta B_{C}/d}\right)$”, is a first order derivative of sCr (or pCysC) vs. time reflecting rate of change in sCr (or pCysC) analogous to the relationship of distance vs. time and velocity. Here, larger falls in circulating biomarker concentrations (consistent with prompt allograft function) produce higher KeGFR values. Increasing circulating biomarker values (negative values of ΔB_c_) produce lower KeGFR values. Thirdly, unlike urinary biomarkers, interpretation of circulating biomarkers is not complicated by transient anuria and the consequent failure to collect urine [13].

Limitations of this study include those underpinning the kinetic formula itself [1], and those in study design. Since B_c_ cannot be collected continuously, the methodology borrows from the ancient mathematical method of exhaustion to produce an estimated mean GFR over time, rather than instantaneous real-time evaluation. Abrupt changes in GFR, *e*.*g*. due to renal vein thrombosis, may not be recognized although similar limitations apply to clearance-based GFR measurements using iothalamate or EDTA [4]. Circulating biomarker concentrations are likely to be most affected by pre-operative dialysis and intra-operative fluid administration immediately after transplantation. This may explain the poorer performance of KeGFR_sCr_ at 4h than at 8h and 12h.

The formula adopts mass balance principles [1,19] stipulating that change in the amount of creatinine (or CysC) in the body over time, is equal to the amount of creatinine generated (or CysC) minus the amount of creatinine (or CysC) excreted. The generation of KeGFR < 0 seen in some patients (*e*.*g*. nine patients for KeGFR_sCr_ at 12h) probably reflects either an underestimate of MaxΔB_c_/d in the affected patients [1] or error in the measurement of the relevant biomarker at one or both times, inherent to all assays.

There are several potential sources of inaccuracy in the estimation of MaxΔB_c_/d. Firstly, the methodology does not account for factors like perioperative muscle wasting affecting creatinine generation, or corticosteroid administration [26] affecting CysC generation. Secondly, Chen, like several authors [19,28], assumed an equivalence of CrCl and GFR. This leads to the inaccurate assumption that any sCr multiplied by the corresponding steady-state eGFR (or pCysC × eGFR) is equivalent and indicates daily creatinine (or CysC) production [1]. Unlike the Cockroft-Gault formula, derived from measured Cr excretion [8,16,29], back calculation from the empirical CKD-EPI formula [14,30] produces lower estimates of creatinine production at higher values of sCr. For consistency, we arbitrarily used the value of sCr (or pCysC) at 4h to estimate creatinine (or CysC) production and B_c_×eGFR. Despite the theoretical limitations, the sensitivity analysis suggested that the receiver operator characteristics of KeGFR were robust to different assumptions of creatinine or CysC production. Thirdly, the one-compartment kinetic model used might modestly mischaracterize the distribution of creatinine or CysC [3,19]. Fourthly, calculations do not account for changing plasma volumes and hence V_d_ [1,8]. To incorporate these factors into calculations would require repeated patient weighing after surgery or very accurate fluid balance charts, strategies with inherent practical limitations and imprecision [3,31].

This is a retrospective analysis. Future prospective studies would ideally obtain pCysC at 0h, allowing evaluation of KeGFR_pCysC_ at 0h–4h; the absence of these results may obscured the possible superiority of KeGFR_pCysC_ at this early time. Despite the weaknesses of current methods of measuring GFR in non-steady state conditions it would be useful to compare measured GFR to KeGFR, particularly since KeGFR_sCr_ yielded higher values and diagnostic cut-offs than KeGFR_sCr_. A further limitation is the modest cohort size. A comprehensive risk model requires external validation and a much larger data set than available here, and this study cannot resolve which of multiple possible clinical risk models [8,29,32] is best. Although the baseline model adjusts for donation after cardiac death in calculating risk of DGF, a meaningful comparison recipients of DCD-kidneys and donation-after-brain-death-kidneys was not possible. This is not an attempt at validation, but rather an exploration of whether a new biomarker, kEGFR, provides independent prediction of outcome. In this proof of concept study, kEGFR independently predicted DGF adding to a validated risk model. It remains necessary to undertake a large multicentre validation study, potentially incorporating more complex patients and greater variation in fluid management and immunosuppressive approaches that impact renal blood flow and clearance.

Two semantic issues warrant attention. Firstly, defining DGF by dialysis requirement has the advantage of simplicity [30], is associated with increased rejection and graft loss [3], underlies the clinical risk prediction model used here [8], and is the most common method [3]. However the definition is arguably subjective, and any future interventional studies should be powered to examine hard endpoints such as long-term graft function and graft-survival. Secondly, because DGF is mainly the consequence of perioperative ischaemia-reperfusion injury, it is debatable whether DGF is “predicted” or simply “detected” before dialysis is initiated. We have used the term “predicted” to emphasise that early identification of risk will facilitate early and appropriate triaging of affected patients.

In conclusion, applying the KeGFRsCr, derived from readily obtainable sCr results, improved DGF prediction. Like sCr, KeGFR can be inexpensively measured, and clinical application would easily facilitated with a web-based calculator. Use of this equation might facilitate trials of early intervention aimed at ameliorating ischemia-reperfusion injury.

## Supporting Information

S1 Table Sensitivity, specificity, and predictive values for dialysis within 1 week of kidney transplant using specific values of sCr, pCysC KeGFR_sCr_ and KeGFR_pCysC_. Key: NPV: negative predictive value; PPV: positive predictive value. Cut-off values shown were those nearest 90% sensitivity, optimised cut-offs, and those nearest 90% specificity.
(DOCX)

S2 Table Sensitivity analysis comparing alternative assumptions in the kinetic estimates of GFR (KeGFR). Comparison of utility of KeGFR formula for prediction of DGF using different estimates of the MaxΔsCr/d, maximal theoretical increase in sCr in 1 day when GFR is zero, and of MaxΔpCysC/d. MaxΔsCr/d was estimated using back calculation from the CKD-EPI formula, and alternatively using the Cockroft-Gault formula, and a fixed value (235 mmol/L/d) for all patients. MaxΔpCysC/d was estimated using back calculation from the CKD-EPI formula, and alternatively using the Sjostrom formula, and a fixed value (3 mg/L/d). Optimal cut-offs were values with the maximal Youden index for prediction of DGF. Key: AUC: area under the receiver operator characteristic curve; NPV: negative predictive value; PPV: positive predictive value.
(DOCX)

S3 Table Sensitivity analysis for prediction of DGF using KeGFR expressed as mL/min. KeGFR_sCr_ (expressed in mL/min) and KeGFR_pCysC_ (mL/min) were produced by using the relevant reference formula (producing KeGFR, expressed in mL/min/1.73m^2^) and multiplying by BSA/1.73 (body surface area, calculated using the formula of Dubois and Dubois: *BSA* = 0.007184×*W* ^0.425^× *W* ^0.725^.) Key: AUC: area under receiver operator characteristic curve. P values listed for difference with reference formula. a: there is no KeGFR_pCysC_ at 4h since no 0h pCysC data were available.
(DOCX)

S4 Table KeGFR prediction of DGF compared with unadjusted eGFR and sCr including deceased and live donor kidneys. Four patients commenced dialysis between 4h and 8h, leaving 78 patients for analysis at 8h and 12h. Key: AUC: area under receiver operator characteristic curve. P values listed for difference with AUC-sCr. a: p > 0.05 for difference with pCysC. b: there is no KeGFR_pCysC_ at 4h since no 0h pCysC data were available. Characteristics of the cohort have been previously presented.
(DOCX)
