# Supplementary material for: Kinetic Estimation of GFR Improves Prediction of Dialysis and Recovery after Kidney Transplantation
Source: PLoS One. 2015 May 4;10(5):e0125669. doi: 10.1371/journal.pone.0125669 (PMC4418565; doi:10.1371/journal.pone.0125669)
Supplement: S1 Table — Key: NPV: negative predictive value; PPV: positive predictive value. Cut-off values shown were those nearest 90% sensitivity, optimised cut-offs, and those nearest 90% specificity. (DOCX) [file pone.0125669.s001.docx]

**S1 Table. Sensitivity, specificity, and predictive values for dialysis within 1 wk of kidney transplant using specific values of sCr, pCysC KeGFR_sCr_ and KeGFR_pCysC_.**

Key: NPV: negative predictive value; PPV: positive predictive value. Cut-off values shown were those nearest 90% sensitivity, optimised cut-offs, and those nearest 90% specificity.
