# Supplementary material for: Kinetic Estimation of GFR Improves Prediction of Dialysis and Recovery after Kidney Transplantation
Source: PLoS One. 2015 May 4;10(5):e0125669. doi: 10.1371/journal.pone.0125669 (PMC4418565; doi:10.1371/journal.pone.0125669)
Supplement: S2 Table — Comparison of utility of KeGFR formula for prediction of DGF using different estimates of the MaxΔsCr/d, maximal theoretical increase in sCr in 1 day when GFR is zero, and of MaxΔpCysC/d. MaxΔsCr/d was estimated using back calculation from the CKD-EPI formula, and alternatively using the Cockroft-Gault formula, and a fixed value (235 mmol/L/d) for all patients. MaxΔpCysC/d was estimated using back calculation from the CKD-EPI formula, and alternatively using the Sjostrom formula, and a fixed value (3 mg/L/d). Optimal cut-offs were values with the maximal Youden index for prediction of DGF. Key: AUC: area under the receiver operator characteristic curve; NPV: negative predictive value; PPV: positive predictive value. (DOCX) [file pone.0125669.s002.docx]

##

## S2 Table. Sensitivity analysis comparing alternative assumptions in the kinetic estimates of GFR (KeGFR).

Comparison of utility of KeGFR formula for prediction of DGF using different estimates of the MaxΔsCr/d, maximal theoretical increase in sCr in 1 day when GFR is zero, and of MaxΔpCysC/d. MaxΔsCr/d was estimated using back calculation from the CKD-EPI formula (1), and alternatively using the Cockroft-Gault formula (2), and a fixed value (235 mmol/L/d) for all patients (3). MaxΔpCysC/d was estimated using back calculation from the CKD-EPI formula (4), and alternatively using the Sjostrom formula (5), and a fixed value (3 mg/L/d) (3). Optimal cut-offs were values with the maximal Youden index for prediction of DGF.

Key: AUC: area under the receiver operator characteristic curve; NPV: negative predictive value; PPV: positive predictive value.

**References**

1. Levey AS. A New Equation to Estimate Glomerular Filtration Rate. *Ann Intern Med* 150:604, 2009

2. Cockcroft DW, Gault H. Prediction of Creatinine Clearance from Serum Creatinine. *Nephron* 16:31–41, 1976

3. Chen S. Retooling the creatinine clearance equation to estimate kinetic GFR when the plasma creatinine is changing acutely. *J Am Soc Nephrol* 24: 877–888, 2013

4. Inker LA, Schmid CH, Tighiouart H, Eckfeldt JH, Feldman HI, Greene T, et al. Estimating Glomerular Filtration Rate from Serum Creatinine and Cystatin C. *N Engl J Med* 367:20–29, 2012

5. Sjöström P, Tidman M, Jones I. Determination of the production rate and non-renal clearance of cystatin C and estimation of the glomerular filtration rate from the serum concentration of cystatin C in humans. *Scand. J. Clin. Lab. Invest.* 65:111–124, 2005
