# Supplementary material for: Kinetic Estimation of GFR Improves Prediction of Dialysis and Recovery after Kidney Transplantation
Source: PLoS One. 2015 May 4;10(5):e0125669. doi: 10.1371/journal.pone.0125669 (PMC4418565; doi:10.1371/journal.pone.0125669)
Supplement: S3 Table — KeGFRsCr (expressed in mL/min) and KeGFRpCysC (mL/min) were produced by using the relevant reference formula (producing KeGFR, expressed in mL/min/1.73m2) and multiplying by BSA/1.73 (body surface area, calculated using the formula of Dubois and Dubois: BSA = 0.007184×W 0.425× W 0.725.) Key: AUC: area under receiver operator characteristic curve. P values listed for difference with reference formula. a: there is no KeGFRpCysC at 4h since no 0h pCysC data were available. (DOCX) [file pone.0125669.s003.docx]

## S3 Table. Sensitivity analysis for prediction of DGF using KeGFR expressed as mL/min.

KeGFR_sCr_ (expressed in mL/min) and KeGFR_pCysC_ (mL/min) were produced by using the relevant reference formula (producing KeGFR, expressed in mL/min/1.73m^2^) and multiplying by BSA / 1.73 (body surface area, calculated using the formula of Dubois and Dubois: $BSA=0.007184\times W^{0.425}\times W^{0.725}$.)

Key: AUC: area under receiver operator characteristic curve. P values listed for difference with reference formula. a: there is no KeGFR_pCysC_ at 4h since no 0h pCysC data were available.
