# Supplementary material for: Kinetic Estimation of GFR Improves Prediction of Dialysis and Recovery after Kidney Transplantation
Source: PLoS One. 2015 May 4;10(5):e0125669. doi: 10.1371/journal.pone.0125669 (PMC4418565; doi:10.1371/journal.pone.0125669)
Supplement: S4 Table — Four patients commenced dialysis between 4h and 8h, leaving 78 patients for analysis at 8h and 12h. Key: AUC: area under receiver operator characteristic curve. P values listed for difference with AUC-sCr. a: p > 0.05 for difference with pCysC. b: there is no KeGFRpCysC at 4h since no 0h pCysC data were available. Characteristics of the cohort have been previously presented. (DOCX) [file pone.0125669.s004.docx]

## Table S4. KeGFR prediction of DGF compared with unadjusted eGFR and sCr including deceased and live donor kidneys.

Four patients commenced dialysis between 4h and 8h, leaving 78 patients for analysis at 8h and 12h (Table 2).

Key: AUC: area under receiver operator characteristic curve. P values listed for difference with AUC-sCr. a: p > 0.05 for difference with pCysC. b: there is no KeGFR_pCysC_ at 4h since no 0h pCysC data were available. Characteristics of the cohort have been previously presented (1).

**Reference**

1. Pianta TJ, Peake PW, Pickering JW, Kelleher M, Buckley NA, Endre ZH. Clusterin in kidney transplantation: novel biomarkers versus serum creatinine for early prediction of delayed graft function. *Transplantation* Epub ahead of print, 2014
